# Chinese Technical Guideline for Deriving Water Quality Criteria for Protection of Freshwater Organisms

**DOI:** 10.3390/toxics11020194

**Published:** 2023-02-19

**Authors:** Zhen-Guang Yan, Xin Zheng, Yi-Zhang Zhang, Zhan-Hong Yang, Quan Zhou, Shu-Hui Men, Jin-Zhe Du

**Affiliations:** 1 State Key Laboratory of Environmental Criteria and Risk Assessment, Chinese Research Academy of Environmental Sciences, Beijing 100012, China; 2 Environmental Standards Institute of Ministry of Ecology and Environment of the People’s Republic of China, Beijing 100012, China; 3 School of Marine Science and Engineering, Qingdao Agricultural University, Qingdao 266061, China

**Keywords:** ecotoxicity data, freshwater organism, technical guideline, water quality criteria

## Abstract

In recent years, China has determined the national goal of “developing national environmental criteria”, thereby promoting the rapid development of environmental quality criteria research in China. In 2017, the Ministry of Ecology and Environment of China (MEEC, formerly the Ministry of Environmental Protection of China) issued the technical guideline for deriving water quality criteria (WQC) for protection of freshwater organisms (HJ 831-2017), and in 2022, they organized the guideline revision and issued an updated version (HJ 831-2022). The primary contents of the revision included the following. The minimum toxicity data requirements were upgraded from 6 to 10, and the species mean toxicity value was replaced by the same effect toxicity value for the criteria calculation. It is now required that the tested organisms must be distributed in China’s natural fresh waters, and the toxicity data of non-native model species will no longer be used. The list of freshwater invasive species in China that cannot be used as test species was added into the guideline. The acute/chronic ratio (ACR) method for the criteria derivation and the extreme value model were deleted, and the provisions for testing the toxicity data distribution were also deleted. The exposure time of the toxicity test of various tested organisms was refined, and the priority of the toxicity data was clearly specified. This paper introduces the framework and specific technical requirements of HJ 831-2022 in detail, including data collection, pre-processing of toxicity data, criteria derivation, fitting models, and quality control. This introduction is helpful for international peers to understand the latest research progress of China’s WQC.

## 1. Introduction

Water quality criteria (WQC) can be divided into aquatic life criteria [1], human health criteria [2], nutrient criteria [3], and sediment criteria [4]. After more than ten years of research [5,6,7,8,9,10,11,12], in 2017, the Ministry of Ecology and Environment of China (MEEC, formerly the Ministry of Environmental Protection of China) issued technical guidelines for deriving the freshwater aquatic life criteria (the technical guideline for deriving WQC for protection of freshwater organisms, HJ 831-2017) [13,14], human health water quality criteria [15], and lake nutrient criteria [16]. This is the first time for China to issue WQC guidelines. Based on the issued aquatic life criteria guideline, China developed the WQC for the protection of freshwater organisms for cadmium [17], ammonia [18], and phenol [19] in 2020. In the process of developing these three criteria, some technical deficiencies were found in the HJ 831-2017. Therefore, in 2022, under the authorization of the MEEC, the Chinese Research Academy of Environmental Sciences took the lead in organizing the revision of HJ 831-2017. This paper introduces the document framework and features of the issued updated guideline (HJ 831-2022) [20].

## 2. The Updated Guideline for Deriving Freshwater WQC

### 2.1. Framework and Overview of the Guideline

According to the Chinese national standards for the development of technical guidelines [21], Guideline HJ 831-2022 consist of 11 parts: the scope of application, reference standards, terms and definitions, the criteria deriving procedure, the scheme formulation, data acquisition, the criteria derivation, quality control, an uncertainty analysis, report preparation, and four appendices (A, B, C, and D).

The guideline stipulates that the species sensitivity distribution (SSD) method proposed in this guideline is applicable to the development of the WQC for a single chemical pollutant and is not applicable for endocrine disruptors and highly bioconcentrated chemicals. This is because the toxic effects of endocrine disruptors are complex [22,23], and it may not be possible to simply use the SSD method to derive its criteria. It may be necessary to establish another guideline for the development of WQC for endocrine disruptors [24]. As for highly bioconcentrated chemicals, the development of the WQC requires the data on the maximum allowable concentration (MAC) of the pollutant in biological tissue [1], but the related data are very scarce. Hence, the determination of the MAC of a pollutant in biological tissue may also require a separate guideline.

In the “reference standards” section, this guideline primarily quotes some standard methods for biological toxicity testing to guide researchers to screen toxicity data or conduct supplementary experiments to obtain toxicity data according to the provisions of the standard methods. Most of the preferred standard methods are Chinese national standards of ecotoxicity test guidelines for species such as fish, daphnia, amphibians, crustaceans, alga, and duckweed. This Guideline also referenced ISO 20666, the ecotoxicity test method of international organizations (water quality determination of the chronic toxicity to *Brachionus calyciflorus* in 48 h), due to the lack of Chinese standards.

The “terms and definitions” section defines some common terms and their definitions used in the process of criteria development, including “water quality criteria, WQC”, “freshwater quality criteria”, “short-term WQC, SWQC”, “long-term WQC, LWQC”, “bioconcentration factor, BCF”, “very bioaccumulative pollutants”, “species sensitivity distribution, SSD”, “median lethal concentration, LC_50_”, “x% effect concentration, ECx”, “lowest observed effect concentration, LOEC”, “no observed effect concentration, NOEC”, “maximum acceptable toxicant concentration, MATC”, “acute toxicity value, ATV”, “chronic toxicity value, CTV”, “acute value for the same effect, AVE”, “chronic value for the same effect, CVE”, “hazardous concentration for x% species, HC_x_”, and “assessment factor, AF”.

From the “criteria deriving procedure” section to the “report preparation” section, the technical requirements of the entire process of the WQC development are specified in detail as follows.

### 2.2. Criteria Deriving Procedure

**Figure 1:**
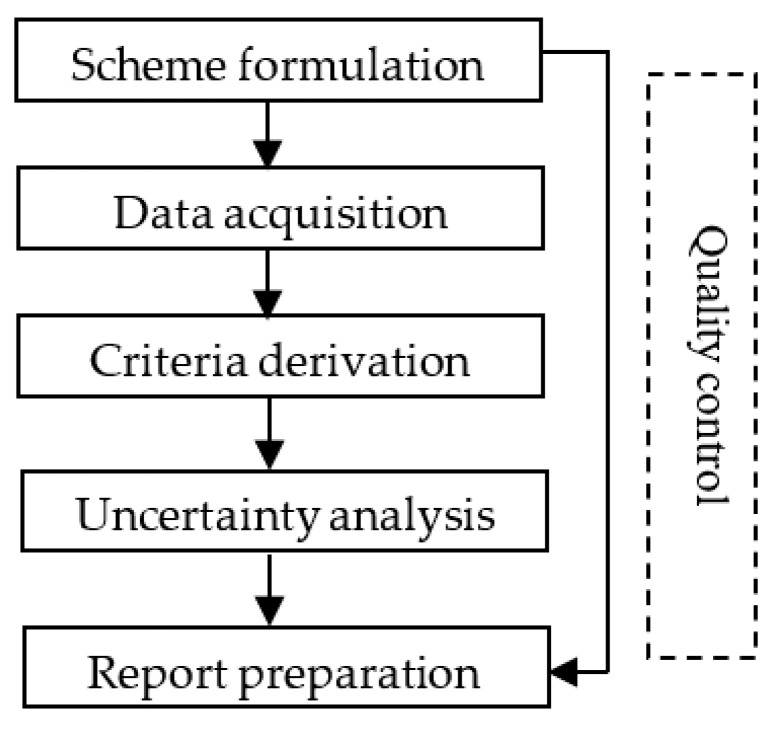
Development procedure of the freshwater WQC.

This guideline divides the criteria development process into five steps (Figure 1) including scheme formulation, data acquisition, criteria derivation, uncertainty analysis, and report preparation. Quality control is performed throughout the criteria development process.

### 2.3. Scheme Formulation

This is the first step of criteria development. In this step, researchers are required to (1) clarify the working requirements of criteria development and understand the relevant background information, such as the physicochemical properties and environmental behavior of the target pollutant, and the research progress of the relevant WQC; (2) determine the source of the relevant data required to derive the criteria, the retrieval method and retrieval plan to obtain these data, and the technical requirements for data screening and evaluation; (3) master the technical points of criteria derivation, including the SSD model-fitting principle, the criteria derivation software, and the determination and expression of criteria. If necessary, the adjustment model of toxicity data also needs be determined. Finally, the researchers should grasp the requirements for the preparation of the criteria technical report and prepare the criteria development plan after consulting the opinions of environmental managers and stakeholders.

### 2.4. Data Acquisition

#### 2.4.1. Data Acquisition Procedure

This step of data acquisition is divided into data collection, data screening, and data evaluation.

#### 2.4.2. Data Collection

Criteria development requires multiple types of data. This guideline specifies that the data to be collected include (1) basic information of the target pollutant, such as name, molecular formula, Chemical Abstracts Service (CAS) number, and uses; (2) inherent physicochemical property data of the pollutant, including melting point, boiling point, solubility, volatility, octanol water partition coefficient, chemical equilibrium constant, and half-life; (3) exposure data of the pollutant in the water environment; (4) ecotoxicity data of the pollutant to Chinese freshwater organisms; (5) information of the test organism including Chinese name, common name, scientific name, classification, living habitat characteristics, and geographical distribution area; and (6) toxicity-related water quality parameters data of the surface water body including temperature, pH, hardness, organic matter content, suspended particle content, monitoring time, and monitoring area or site information.

The data used to derive the WQC can be collected from a variety of sources including (1) the ecotoxicity database; (2) published literature or reports; (3) relevant data released by the government; (4) data from other sources judged by experts to be reliable; and (5) toxicity data from supplementary experiments with Chinese resident species.

#### 2.4.3. Data Screening

##### Test Organism Screening

The test species used for deriving the criteria should (1) live in Chinese freshwater bodies. Invasive alien species cannot be used, and rare or endangered species should be used according to the related law and regulations; (2) be able to be domesticated, propagated, and have enough individuals for toxicity experiments in the laboratory; (3) have high sensitivity and consistent toxicity reaction to pollutants; (4) have standardized toxicity test endpoints and methods; (5) maintain stable genetic traits during artificial domestication and reproduction; and (6) be ensured to not have been exposed to the target pollutant before. Finally, unicellular animals and microorganisms (except unicellular algae) cannot be used.

##### Toxicity Data Screening

First, the guideline specifies the acute and chronic data to be used for the criteria development. The acute toxicity data are generally divided into two categories: growth data (e.g., body weight, body length, growth rate, and biomass) and survival data (e.g., survival rate and mortality rate); and the related toxic effect can be indicated using EC_50_ and LC_50_. The chronic toxicity data are generally divided into three categories: growth data (e.g., body weight, body length, growth rate, biomass), reproduction data (e.g., incubation rate, incubation time, and sex ratio), and survival data (e.g., survival rate and mortality rate); their relevant effect indicators include MATC, EC_10_, EC_20_, NOEC, LOEC, EC_50_, and LC_50_.

For the screening of toxicity data, the guideline stipulates that researchers should consider various factors that may affect data quality, including experimental design, test chemical, test organism, experimental condition, and data analysis. In addition, the priority of toxicity data in different situations is also specified.

For the test design, first, the test shall be in accordance with national or international standard toxicity test methods. The relevant documents of other standard organizations or countries can also be referred to; otherwise, the test design shall be described in detail. Second, a blank control group is essential for the test, and a positive control group shall also be established if necessary. The use of cosolvents or dispersants should be avoided as much as possible. If it is necessary to use, a solvent control group shall be established, with the concentration generally not exceeding 0.1 mL/L, and the concentration shall be consistent in all containers. In addition, the cosolvent or dispersant shall not have a significant effect on the test results. Third, the concentration of the test group shall be established according to the requirements of the standard toxicity test method. The concentration interval coefficients of the acute and chronic toxicity tests generally do not exceed 2.2 and 3.2, respectively. Finally, in order to ensure the reliability of the test results, the toxicity test requires the design of a certain number of parallel groups.

The experimental reagents should have accurate names and CAS numbers. When the reagent is an inorganic salt, the chemical form of the reagent substance of the experimental results should be stated. Moreover, the reagent purity should generally be greater than 95%; otherwise, the reliability of the experimental results needs to be judged by an expert, and the experimental data should be corrected according to the reagent purity, or the measured concentration should be used.

For the test organism, the necessary information should include the scientific name, life stage, and source (e.g., laboratory, breeding farm, or the wild), and those obtained in the wild should indicate the specific geographic location of the species. Before the experiment begins, some period of acclimatization of the test organisms under the experimental conditions is recommended. The mortality rate of the standard test organisms during acclimatization should meet the requirements of the standard test method (generally 5%). The maximum mortality rate of non-standard test organisms during acclimatization cannot exceed 10%.

The test exposure conditions are very important for judging whether toxicity data are reliable. Therefore, this guideline specifies several technical requirements for this. (1) For a test substance with high volatility and degradability, its concentration should be determined during the toxicity test. For other substances, the nominal concentration can be used, but when no cosolvent is used, the nominal exposure concentration of the test substance should be lower than its solubility in water. (2) The test system should be suitable for the survival of the tested organism, and the water quality should be stable within a certain range according to the survival requirements of the tested organism. The dissolved oxygen saturation of the test solution should be greater than 60%. (3) The bioburden of the toxicity test system should comply with the provisions of the toxicity test standard. (4) Generally, feeding is not allowed during an acute test unless there is evidence that feeding will not affect the final test results. (5) The chronic toxicity test generally adopts the flow-through or renewal exposure types, and the acute toxicity test can also adopt the static type. Microalgae are generally suitable for the static or renewal type. (6) For the acute toxicity test, the exposure time of rotifers is 24 h, that of daphnia and chironomids is 48 h, and that of other species is 96 h. The suitable exposure time of aquatic plants is 96 h. (7) For chronic toxicity test, the exposure time of rotifers is not less than 48 h, and that of other animals is not less than 21 d or covers a sensitive life stage. The suitable exposure time of aquatic plants is not less than 21 d or at least one generation.

Prior to the analysis of test data, the guideline specifies several principles for verifying the validity of data. It stipulates that the variation range of the biological parameters (e.g., algae reproduction speed and animal death or activity inhibition rate) of the control group should meet the requirements of the test standard. The experimental data should be analyzed with appropriate statistical methods for different test endpoints, and the experimental results should meet the statistical requirements. When the experimental data of the same toxicity endpoint of the same species differ by more than 10 times, the outliers should be eliminated. When outliers cannot be determined, all of the relevant data should be discarded.

The guideline specifies the priority of the toxicity data obtained under different circumstances. Flow-through experimental data are preferred to semi-static data over static data. Concentration-measured toxicity data are preferred to nominal concentration data, and toxicity data for sensitive life stages have priority over that for insensitive life stages. The priority of chronic data of the same species is ranked as follows: MATC > EC_20_ > EC_10_ = NOEC > LOEC > EC_50_ > LC_50_. In addition, the full-life-cycle toxicity data take precedence over partial-life-cycle data over single-life-stage data.

##### Toxicity Data Evaluation

The guideline specifies that toxicity data should be evaluated in terms of test methods, experimental procedures, and experimental results. According to the evaluation results, the toxicity data can be divided into the following four categories: (1) unlimited reliable data (the data generation process is fully consistent with the international or national standard test methods or industry technical standards); (2) limited reliable data (the data generation process is not fully consistent with the standard test methods, but the experimental procedures are informative and reliable, and there is sufficient evidence to prove that the data are acceptable); (3) unreliable data (the data generation process conflicts with the standard testing methods, there is not sufficient evidence to prove that the data are acceptable, and the experimental process is not convincing); and (4) uncertain data (the data lack enough experimental details to judge the reliability of the data). Unlimited reliable and limited reliable data can be used for the WQC derivation.

In the guideline, the minimum toxicity data requirement (MTDR) for the WQC derivation is also specified. The test organisms should include at least ten species from three trophic levels (producer, primary consumers, and secondary consumers): one Cyprinidae fish, one non-Cyprinidae fish, one zooplankton, one benthic macroinvertebrate, one amphibian or other aquatic animal belonging to different phyla from the above animals, and one phytoplankton or aquatic vascular plant. In addition, the criteria derivation for herbicides should include at least the toxicity data of one phytoplankton and one aquatic vascular plant, and the criteria derivation for pesticides should include the toxicity data of aquatic insects.

When the quantity of acceptable toxicity data that can be used to derive the WQC is insufficient, the guideline recommends resident freshwater organisms for the ecotoxicity testing that includes 36 species of freshwater animals and 10 species of aquatic plants.

### 2.5. WQC Derivation

#### 2.5.1. WQC Derivation Procedure

**Figure 2:**
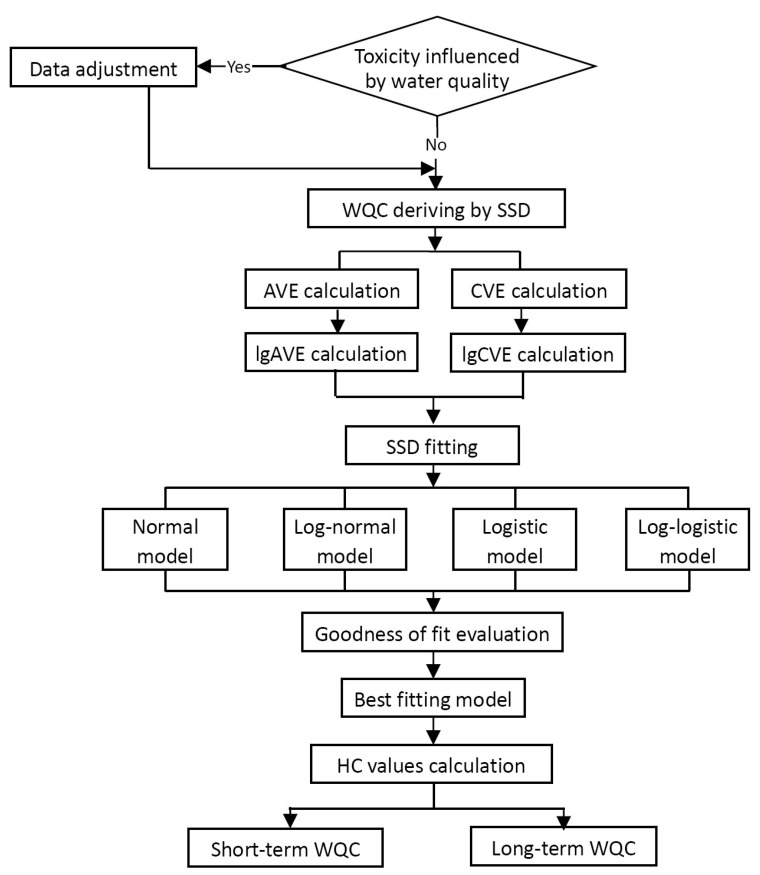
Derivation procedure of the freshwater WQC.

The guideline stipulates that the SSD method is used to derive the WQC, and the derivation procedure is shown in Figure 2.

#### 2.5.2. Toxicity Data Pre-Processing

##### Analysis of the Influence of Water Quality on the Toxicity of Pollutants

First, researchers need to analyze the impact of water quality parameters on the toxicity of pollutants. The water quality parameters (e.g., temperature, hardness, and pH) should be used as independent variables, and the corresponding toxicity values should be used as dependent variables for the correlation regression analysis. When the water quality has a significant influence on the toxicity of the target pollutant, the toxicity data must be adjusted according to the water quality conditions before they can be utilized for the WQC calculation. After that, the AVE, lgAVE, CVE, and lgCVE are calculated.

##### AVE Calculation

When calculating the AVE, EC_50_ is utilized as the growth ATV and LC_50_ as the survival ATV, and these are substituted into Equation (1) to calculate the growth AVE and survival AVE, respectively, of each species.

(1) $${\mathrm{AVE}}_{i,k}=\sqrt[{m}]{{\mathrm{ATV}}_{i,k,1}{\times \mathrm{ATV}}_{i,k,2}{\times \ldots \times \mathrm{ATV}}_{i,k,m}}$$

where AVE is the acute value of the same effect; i represents a certain species; k represents the type of acute toxic effect that is generally divided into growth and survival; m is the number of ATV; and ATV is the acute toxicity value.

The smaller value of the growth AVE and survival AVE is selected for subsequent calculation. If only one AVE is obtained, it is directly used for the subsequent calculation.

##### CVE Calculation

When calculating the CVE, the guideline stipulates that the MATC shall be calculated using Equation (2) first.

(2) $${\mathrm{MATC}}_{i,z}=\sqrt{{\mathrm{NOEC}}_{i,z}{\times \mathrm{LOEC}}_{i,z}}\;$$

where the MATC is the maximum acceptable toxicant concentration; NOEC is the no observed effect concentration; LOEC is the lowest observed effect concentration; i represents a certain species; and z represents the type of toxic effect.

According to the different effect (growth or reproduction), the chronic toxicity data are utilized as the growth CTV or the reproduction CTV, respectively, and LC_50_ is utilized as survival CTV, and these are substituted into Equation (3) to calculate the CVE of different species.

(3) $${\mathrm{CVE}}_{i,j}=\sqrt[{n}]{{\mathrm{CTV}}_{i,j,1}{\times \mathrm{CTV}}_{i,j,2}{\times \ldots \times \mathrm{CTV}}_{i,j,n}}$$

where the CVE is the chronic value of the same effect; i represents a certain species; j represents the type of chronic toxic effect; n represents the number of CTVs; and CTV represents the chronic toxicity value. If multiple CVE are obtained, the smallest CVE is utilized for subsequent calculations. If only one CVE is obtained, it is directly used for the subsequent calculations.

##### Logarithmic Conversion

For the logarithmic conversion of the toxicity data, the guideline stipulates that AVE and CVE should be utilized as the common logarithms, and the obtained lgAVE and lgCVE must be all positive values; otherwise, the AVE and CVE values should be transformed first.

#### 2.5.3. Model Fitting and Evaluation

##### Calculation of the Cumulative Frequency

Before model fitting, it is necessary to calculate the cumulative frequency. First, the lgAVE and lgCVE are ranked from small to large, and the rank with the lowest toxicity value is 1. If two or more toxicity values are the same, they should be randomly ranked into consecutive ranks. The acute and chronic cumulative frequencies of the species are then calculated using Equation (4).

(4) $$F_{R}=\frac{\sum_{1}^{R}f}{\sum f+1}\times 100\%$$

where FR is the cumulative frequency; R is the rank; and f is the number of species corresponding to the rank of a certain toxicity value.

##### Model Fitting

With lgAVE and lgCVE as the independent variable x and the corresponding FR as the dependent variable y, the SSD model fitting is performed using the normal distribution model, the log normal distribution model, the logistic model, and the log logistic model. It is recommended to use the “National Ecological Environment Criteria Calculation Software, the species sensitivity distribution (version 1.0)” (download website: https://www.mee.gov.cn/ywgz/fgbz/hjjzgl/mxrj/202203/t20220304_970658.shtml, accessed on 16 February 2023) developed by the State Key Laboratory of Environmental Criteria and Risk Assessment for the calculation.

##### Evaluation of the Fitting Results

The results of model fitting are evaluated according to the goodness of fit. The evaluation parameters are the root mean square (RMSE, the smaller the better) and the *p*-value (A–D test, *p* should be greater than 0.05). The best-fitting model should be determined according to the evaluation result combined with expert judgment.

#### 2.5.4. Determination of the Hazardous Concentrations

According to the best-fitting model, when y is a certain FR value, the corresponding x value can be calculated, and then, the x or its reversed transformed value is the corresponding hazard concentration (HC). The guideline stipulates that the long-term and short-term HC_5_, HC_10_, HC_25_, HC_50_, HC_75_, HC_90_, and HC_95_ corresponding to Y values of 5%, 10%, 25%, 50%, 75%, 90%, and 95%, respectively, should be calculated.

#### 2.5.5. WQC Calculation

By dividing the long-term or short-term HC5 by the assessment factor (AF), the corresponding long-term or short-term WQC can be obtained. The value of AF is determined based on the number of toxicity data. When the number of acceptable AVE or CVE is higher than 15, the AF value is set at two. When the number of acceptable AVE or CVE is not higher than 15, the AF value is set at three. Under special circumstances, such as if the proportion of algae toxicity data exceeds 50%,s or the fitting result of the SSD curve tail is poor, the value of AF is determined by experts.

#### 2.5.6. Determination and Expression of the WQC

The guideline make the following provisions for the final determination and expression of the WQC: (a) the WQC derived according to this guideline includes short-term and long-term criteria; (b) it should be ensured that the short-term and long-term WQC are less than the minimum AVE and CVE, respectively, of all important freshwater species with high economic value or outstanding ecological significance; otherwise, the minimum AVE or CVE of the most sensitive important species should be utilized as the criteria values; (c) the criteria value is determined based on information such as the pollutant toxicity and instrument detection limit. Generally, two to four significant figures are reserved, and the unit is μg/L or mg/L; (d) the WQC expression includes the criteria values, HC_5_, and AF. If the toxicity of the target pollutant is affected by water quality, the expression should also include the water quality information corresponding to the criteria.

### 2.6. Quality Control

The guideline specifies how quality control should be conducted at each stage of WQC development. For example, the data retrieval staff should be trained in data retrieval knowledge and skills. After the data screening is finished, the data screening staff should comprehensively display the obtained various data information and explain the reasons for all data that are excluded. The key data that affect the criteria value should be verified by at least two people on the source and reliability of the data. People conducting supplementary toxicity experiments should be trained in standard test methods, and additionally, relevant staff should be trained in criteria derivation methods.

### 2.7. Uncertainty Analysis

The guideline requires a qualitative analysis of the uncertainty in criteria development. The generation of uncertainty involves data acquisition, model selection, criteria derivation, and other related steps including but not limited to data sources, retrieval schemes, data screening and evaluation, representativeness of tested species, toxicity data adjustment, SSD fitting model evaluation, and determination of the AF value.

### 2.8. Report Preparation

A recommended framework for WQC technical report is given in the guideline. The primary content of the technical report includes relevant research progress at home and abroad, environmental issues of the target pollutants, data collection and screening, and the criteria derivation process.

### 2.9. Appendices

The guideline has four appendices. Appendix A is a reference table for data collection in which various data to be collected during the process of development of WQC are listed. Appendix B shows a list of 46 common sensitive species distributed in Chinese freshwater bodies to be recommended for the toxicity test. Appendix C is a list of 12 freshwater invasive species in China based on the list of Chinese invasive species issued by the MEEC, and these invasive species cannot be used as test organisms for criteria development. Appendix D specifies the outline and specific requirements in preparation of the criteria technical report.

## 3. Discussion

Developed countries have been studying WQC for more than half a century and have established a relatively complete system of technical methods [1,25,26,27,28]. When China established its own WQC technical methods, it referred to the experience of some developed countries, such as the toxicity data screening and model fitting. However, China’s guideline differs in the specific screening method of toxicity data and the model selected for SSD fitting. In addition, compared with developed countries, China’s WQC technical guideline have added quality control, an uncertainty analysis, and report preparation.

The MTDR is one of the important technical requirements for criteria development. For example, the United States (U.S.) guideline requires the toxicity data of eight families from three phyla and one aquatic plant to derive the criteria, while other countries require fewer toxicity data, i.e., five to six toxicity data of different species [25,26,27,28]. In 2017, when China first formulated the WQC guideline for the protection of freshwater organisms (HJ 831-2017), the MTDR was six species from three trophic levels. In this revision, the HJ 831-2022 upgraded the MTDR to 10 species, which is basically higher than the requirements of most countries. In addition, considering that Cyprinidae is one of the major resident fish in China, it is clearly stated that toxicity data for at least one Cyprinidae should be obtained. In addition, considering that plants and animals are exposed to pollutants together in aquatic ecosystems, the toxicity data of animals and plants are put together when performing the SSD fitting. This is different from the U.S. guideline but is consistent with the European Union (EU) methods.

As for the selection of test species, considering the harm of invasive species to the natural ecosystem, it is clearly stated that the toxicity data of invasive species cannot be used to derive the criteria. This point is not clearly specified in HJ 831-2017. In addition, a list of native test species for the development of China’s WQC is recommended in the guideline. Regarding the test organisms for criteria development, only the U.S.s recommends some North American test organisms for WQC development in their guideline [1]. However, the U.S. guidelines do not specify how the recommended test species are screened. The recommendations of resident test organisms in the Chinese guideline are based on species sensitivity analysis [29,30,31,32,33,34,35]. The 46 recommended resident test organisms (36 species of freshwater animals and 10 species of algae or freshwater plants) are all Chinese freshwater organisms that are generally sensitive to pollutants. In addition, the guideline also points out that other suitable Chinese freshwater organisms can also be used for criteria development.

The exposure time of the toxicity test for test organisms in criteria development is a relatively difficult issue to determine, especially for phytotoxicity tests. HJ 831-2017 stipulates that the exposure time of the acute toxicity test shall not exceed four days, and the exposure time of the chronic toxicity test shall be more than 21 days. In fact, there is no clear time limit for acute and chronic exposure for plants. The exposure time of toxicity experiments specified in this guideline is primarily determined based on the exposure time specified in international standard test methods. For animals, the acute exposure time is 24 h for rotifers [36], 48 h for Daphnia and Chironomid [37], and 96 h for other organisms such as fish [37]. The chronic exposure time is 48 h for rotifers [38] and more than 21 days [37] or across a sensitive life stage for other animals [37] (such as in the toxicity test for fish early-life stages). For plants, the acute exposure time is defined as 96 h, and the chronic exposure time is defined as greater than 21 days or one generation (such as for phytoplankton [37]). In this way, the revised technical guideline further refined the provisions on exposure times of the toxicity test. The exposure time of toxicity test in the U.S. guideline is different, and the acute exposure time is generally 48 h for Daphnia and Chironomidae or 96 h for other organisms, while the chronic exposure time is relatively variable [1]. In contrast, the Chinese guidelines on exposure time are relatively simple, which is to make the guideline easier to understand and implement by researchers at this stage.

As for the toxicity test endpoint, the acute toxicity data specified in the Chinese guideline are the same as that of developed countries, i.e., LC_50_ or EC_50_. For the endpoint of the chronic toxicity data, the NOEC is used in the EU [28], the LOEC is used in Canada [25], and the MATC is used in the U.S. [1,39]. Considering that the determination of the NOEC and LOEC depends on the setting of the pollutant concentration in the experimental design, there is a certain degree of uncertainty. Therefore, the Chinese guideline stipulates that the MATC (equals to the geometric mean of the NOEC and LOEC) is preferred for chronic toxicity data. When the MATC is not available, the NOEC and LOEC are used in sequence.

As for the fitting model of the criteria derivation, the SSD method is popular. However, different countries use different fitting functions. For example, the U.S. uses log-triangular function, while the EU uses log-logistic or other functions [40]. The HJ 831-2017 stipulates the use of the normal, log-normal, logistic, log-logistic, and extreme value functions for fitting and then determining the final function by comparing the goodness of fit. We believe that it is more conducive to obtain a credible criterion value by selecting the final model through such comparative analysis. However, during the development of the WQC for ammonia, it was found that the extreme value function did not fit well in some cases. This model was therefore abandoned in the revision version of the guidelines.

## 4. Conclusions

The present paper introduces the updated version of the WQC Guideline in China, which was recently revised for the protection of freshwater aquatic organisms in China, running through five major parts: the development procedure, data collection and screening method, selection of fitting models, the preparation of technical report, and quality control. This revision of China’s WQC guidelines has made significant progress and enhances the protection of freshwater organisms, especially in the revised provisions, as the MTDR was upgraded to 10 species, and the data of non-native model species are no longer used. The same effect toxicity value was used to derive the criteria, and the test of the normal distribution of toxicity data was canceled. The priority of toxicity data was clearly provided, and the exposure time of the toxicity test was specified in detail according to the international standard toxicity test guidelines. After this revision, the characteristics of China’s criteria guideline have been further enhanced. In addition, these revisions and relevant considerations in the revision can provide reference for the study of WQC methodology.
